# “Autism research is in crisis”: A mixed method study of researcher’s constructions of autistic people and autism research

**DOI:** 10.3389/fpsyg.2022.1050897

**Published:** 2022-11-24

**Authors:** Monique Botha, Eilidh Cage

**Affiliations:** Division of Psychology, Faculty of Natural Sciences, University of Stirling, Stirling, United Kingdom

**Keywords:** autism, dehumanization, objectification, stigma, participatory research

## Abstract

**Introduction:**

While not all autism research is ableism, autism researchers can be ableist, including by talking about autistic people in sub-human terms (dehumanization), treating autistic people like objects (objectification), and making othering statements which set autistic people apart from non-autistic people, *and* below in status (stigmatization).

**Method:**

This mixed-method study aimed to investigate how autism researchers construct autistic people and autism research, and to investigate whether including autistic people more in research relates to lower ableism in narratives about autistic people. We used a survey with autism researchers (*N* = 195) asking five open-ended questions about autism and autism research, as well as demographics, career length, contact with autistic people (familial and non-familial) and degree to which researchers involve autistic people in their research. We used content analysis to categorize narratives used by autism researchers and cues for ableism (dehumanization, objectification, and stigmatization). We then used binary-logistic regression to identify whether narrative or higher inclusion of autistic people predicted fewer ableist cues, controlling for career length and connections to autistic people.

**Results and discussion:**

Using medicalized narratives of autism predicted higher odds of ableist cues compared to employing social model or neutral embodiment narratives. Greater inclusion of autistic people in research predicted significantly lower odds of ableist cues, while controlling for other contact with autistic people and career length. Next, we used reflexive thematic analysis to analyze researcher’s perceptions of autistic people and autism research. Narratives reflected core ideological disagreements of the field, such as whether researchers consider autism to be an intrinsic barrier to a good life, and whether researchers prioritize research which tackles “autism” versus barriers to societal inclusion for autistic people. Instrumentality (a form of objectification) was key to whether researchers considered a person to have social value with emphasis revolving around intellectual ability and independence. Lastly, language seemed to act as a tool of normalization of violence. Researchers relied on an amorphous idea of “autism” when talking about prevention or eradication, potentially because it sounds more palatable than talking about preventing “autistic people,” despite autism only existing within the context of autistic people.

## Introduction

A progressive year-on-year growth has occurred in the amount of literature published about autism, with a minimum of 18,490 autism research papers published over a ten-year period (2005–2014; Sweileh et al., 2016). In 2019 and 2020 alone, over 7,000 autism research papers were published each year Kirby and McDonald, 2021. Most literature has focused on molecular genetics which seeks to “explain” autism (Sweileh et al., 2016). However, there are core disagreements at the heart of autism research which present considerable challenges, including, but not limited to: the ontology of autism (what the nature of “autism” is; e.g., Feinstein, 2011; Nadesan, 2013; Milton and Timimi, 2016; Hollin, 2017; Chapman, 2020); whether autism is a unitary construct (e.g., Happé et al., 2006; Hollin, 2017); the epistemology of autism research (who creates and what constitutes reliable knowledge about autism or autistic people; e.g., Frith and Happe, 1999; Milton, 2014; Gillespie-Lynch et al., 2017; Hens et al., 2019); what language is most appropriate and most closely reflects the “reality” of autism (e.g., Kapp et al., 2013; Kenny et al., 2016; Bury et al., 2020; Botha et al., 2021); and research funding priorities (e.g., Pellicano et al., 2014; Roche et al., 2020). It has been argued that one reason autism research holds such a particular interest is that, despite significant financial investment, it is a deeply uncertain and contested field (Hollin, 2017).

### The ontology of autism—disease, disorder, disability, difference?

There are disagreements about whether autism should be classified as a disease, disorder, disability, identity, or difference (or a combination of these, e.g., such as differences which amount to disability) (Kapp et al., 2013). The history of the diagnostic category of “autism” is a complicated one. “Autism” was first clinically described as a discrete medical diagnosis in the 1940s by both Leo Kanner (in English) and Hans Aspergers (in German (translated into English only in the 1980s) (Kanner, 1943; Asperger, 1944). Between the 1940s and 1960s descriptions in the literature characterized autistic people as “children, who from early life showed extreme withdrawal” or “profound aloneness,” as “[having] disability in forming “usual” relationships with people,” children who displayed an “insistence on sameness,” “stereotyped movement” and that the “disturbance results in severe characteristic difficulties of social integration” (Kanner, 1943). Later, it would be argued that Kanner plagiarized the work of Hans Asperger, and further, that Asperger himself is said to have plagiarized the work of Russian psychologist, Sukhareva, in 1925 (Silberman, 2015; New and Kyuchukov, 2022).

There has also been a rapid evolution in how autism is conceptualized by researchers (Cashin, 2006), yet clinicians diagnosing autism (according to the DSM-5) seek to identify “persistent deficits in social communication and social interaction across contexts”; including “deficits in social-emotional reciprocity,” “deficits in non-verbal communicative behaviors used for social interactions,” and “deficits in developing and maintaining relationships,” plus “restricted, repetitive behavior” (APA, 2013). Thus, the category which has been rooted in Psychiatry and medicine as a disorder affecting social communicational development still exists as such. This approach aligns with the medical model of disability, positioning disability within the person, as something that could be “fixed” or “cured” (Smart, 2006). Thus, the gold-standard “treatment” of autism focuses on the remediation of autistic symptomology (Walsh, 2011).

Often in opposition to these ideas of autism as a disorder or disease, the neurodiversity and autistic rights movements have positioned autism as combinations of disability, difference, and identity (Sinclair, 1993; Singer, 2017; Bertilsdotter Rosqvist et al., 2020; Kapp, 2020). The term “neurodiversity” describes the idea of variations of the brain and distributed ways of functioning being a fundamental constituent of human biodiversity (Singer, 2017). Both the autistic rights and neurodiversity movement are broad, with people having varying views about whether autism has a biological nature, is socially constructed, or constitutes differences, disability, and/or identity. Most accounts have a degree of realism, describing autism as *mostly* socially-constructed with some shared biological underpinning whose meaning is located within time and place (Hacking, 2001; Nadesan, 2013). Further, these movements focus on the environments and society surrounding autistic people, aligning with the social model of disability (den Houting, 2019). In this context, disability occurs when there is a lack of fit between the person and the environment, with a lack of accommodation for variation (Andrews, 2017).

Given these different approaches, autism researchers are likely to view and research “autism” in different ways dependent on their training and discipline, either focusing more inward on the autistic person, or more outward on social contexts surrounding autistic people. For example, a researcher with a medical model orientation might be more likely to research “treatments” or “interventions” to change autistic behaviors so they conform to non-autistic people’s standards or normative ideas of what constitutes adequate functioning. On the other hand, a researcher with a social model orientation might be more likely to research ways in which environmental adaptations could benefit autistic sensory experiences, good quality of life, or how attitudes toward autistic people shape life for autistic people.

### The epistemology of autism: What should we know, how should we know, and who should be heard?

In addition to medical or social model orientations, autism researchers have power in determining *what* we know about autism and *how* we know it. There is a gap between which autism research topics get funded and topics which are priorities for autistic people and their families. For example, most funding goes toward “basic science” topics (brain, behavior, genetics, causes) [Singh et al., 2009 (USA); Pellicano et al., 2014 (UK); den Houting and Pellicano, 2019 (Australia)]. Further, most autism research focuses on children, even though autistic children grow up to become autistic adults (Kirby and McDonald, 2021). However, it has become apparent that funded autism research does not tend to focus on the priorities of the autistic or wider autism community, which focuses on topics such as post-diagnostic support, mental and physical health, improving public understanding, and improving access to services (Pellicano et al., 2014; Roche et al., 2020). Further, even in terms of *who* is sharing their views on where autism research funding should focus, a systematic review of autism research priorities studies found that only 9% of participants were autistic (Roche et al., 2020). Instead, family members accounted for 61%, while professionals accounted for 24%—meaning a large amount of priority setting rests largely in other (typically non-autistic) people’s hands.

Beyond this gap in priorities and funding, the process of *doing* autism research is also contested. Autism research has had a history of conducting research “on” autistic people and not with them. This approach is opposed to the participatory and citizen-led approach that neurodiversity, critical disability studies, and critical autism studies movements have advocated for—“Nothing About Us Without Us” (Charlton, 2004; Milton, 2014; Bertilsdotter Rosqvist et al., 2020). While some non-autistic researchers are considering more participatory means of doing research, actively involving autistic people as researchers themselves (Chown et al., 2017; Fletcher-Watson et al., 2019), others, however, also express skepticism or misunderstandings about participatory research (Pellicano et al., 2014; den Houting et al., 2021). Furthermore, there have been considerable debates on the place of autistic people in the creation of autism knowledge, with some arguing that autistic people lack epistemic authority (Frith and Happe, 1999; Hens et al., 2019) and there has been a history of the relegation of first-hand accounts as biased or inaccurate by the nature of the fact that autistic people wrote them (Botha, 2021).

### Ableism in autism research

Not only do researchers have power in determining *what* is researched, *who* is involved in the research and *how* knowledge is constructed about autistic people, they also have a role in the language used to talk about autistic people. While there are many ways of talking about autistic people, some are more ableist than others, and further stigmatize or marginalize autistic people (Bottema-Beutel et al., 2020). Ableism can be defined as:

“*A system that places value on people’s bodies and minds based on societally constructed ideas of normality, intelligence, excellence, desirability, and productivity. These constructed ideas are deeply rooted in anti-Blackness, eugenics, misogyny, colonialism, imperialism and capitalism. This form of systemic oppression leads to people and society determining who is valuable and worthy based on a person’s language, appearance, religion and/or their ability to satisfactorily [re]produce, excel and “behave.” You do not have to be disabled to experience ableism*” (Lewis, 2021).

We can see ableism in the way in which autistic people are described. Others have highlighted that the language used to describe autistic people is often dehumanizing (Gernsbacher, 2007; Cowen, 2009; Rose, 2020; Botha, 2021), objectifying (Botha, 2022), or stigmatizing (Bottema-Beutel et al., 2020), and reflects underlying beliefs about the inherent “otherness” of autistic people.

We define dehumanization as “the denial of full humanness to others, and the cruelty and suffering that accompany it” (Haslam, 2006; p. 1). Dehumanization includes denying a group the experience of complex emotions (Leyens et al., 2000), community or identity (Kelman, 1973), excluding a group from shared moral boundaries (Opotow, 1990) and/or denying “uniquely human” (e.g., civility, moral sensitivity or maturity) or “human nature” (e.g., emotional responsiveness, interpersonal warmth or agency) attributes to a group (Haslam, 2006). Research has shown that autistic people are dehumanized by non-autistic people (Cage et al., 2018). This dehumanization may extend into research itself (Gernsbacher, 2007; Yergeau, 2018; Luterman, 2019; Rose, 2020; Botha, 2021). Autistic people have been compared to non-human animals and described as less domesticated than non-autistic people, described as lacking in agency, rationality, epistemic authority, the ability to form community or share culture (see Botha, 2021, for an overview). Accounts from autistic people detail experiences of autism research as othering, dehumanizing, and full of ableism (Luterman, 2019; Rose, 2020; Botha, 2021; Michael, 2021), with autism research determined to establish the inferiority of autistic people (Yergeau, 2018). Additionally, it has been claimed that research creates and fosters stereotypes about autistic people which then invade social discourses about autistic people (Gernsbacher, 2007).

Objectification means to treat a person as an object (Nussbaum, 1995) and was originally conceptualized as the presence of one or more of the following: instrumentality (the objectified being used as a tool for the objectifier’s purpose), fungibility (the objectified is perceived as interchangeable with others like them or objects of other types), violability (the objectified is treated as lacking in boundary integrity or as something acceptable to harm or damage), denial of autonomy (the object is treated as lacking in autonomy or self-determination), inertness (the object is treated as lacking agency or activity) or the denial of subjectivity (the experience, feelings, or wishes of the objectified need not be taken into account). It has been argued that objectification requires someone to treat the objectified both as a tool (through instrumentality), while *also* failing to recognize or actively denying other aspects of the others personhood—such as agency, subjectivity, or experiences (LaCroix and Pratto, 2015).

Autistic people are often objectified in autism research (Botha, 2022), whereby the personhood of autistic people relies on their perceived utility to society which is measured through traditional productivity and notions of independence. Researchers have debated whether or not autistic people are “unaffordable” to society, which is predicated on “intelligence and industry” (Tantam, 2009): “However, it is only a few people with ASD [autism spectrum disorder] who combine originality with high levels of intelligence and industry who are likely to make a sufficiently sustainable, salient contribution that their absence might be considered unaffordable” (p. 219). Further, a sign of potential objectification can be inferred from the fact that in a review of 150 intervention studies on autistic people, only 7% measured or reported adverse events (such as harm) (Bottema-Beutel et al., 2021) meaning autistic people might be considered violable (something which inflicting harm upon is permissible).

### Participatory research methods

There is a growing tradition of involving autistic people in research including in the design, collection, interpretation of data, and dissemination of results. This involves working in a collaborative and open manner alongside autistic people and their families. This approach aims to disrupt the *status quo* of doing research *on* autistic people, to doing research *with* them. There are some good examples of how this is done (Nicolaidis et al., 2015; Fletcher-Watson et al., 2019; Pellicano et al., 2021; Pickard et al., 2022), but perhaps one of the most powerful examples is AASPIRE (Academic Autism Spectrum Partnership in Research and Education). AASPIRE works with autistic people and their families, using power-sharing methods to identify priorities and conduct research projects (such as the creation of healthcare toolkits for autistic people), and have produced guidelines for the inclusion of autistic people (Nicolaidis et al., 2019). Working in this method may disrupt the ableism which proliferates autism research traditionally.

### The present study

Considering the above points, it is important to understand both how researchers construct autistic people and their views on autism research. Autism research is a potentially powerful tool in improving—or restricting—autistic people’s lives. Research and science, which is often positioned as “objective,” holds inherent power despite the fact that it can often reflect social and cultural norms and reproduce inequality (Fondacaro and Weinberg, 2002). Further, researchers are responsible for deciding where large amounts of research funding are directed, by applying for it for their own work and in peer-reviewing others’ applications. Additionally, constructions of autistic people by researchers have leaked into the social and cultural descriptions of autistic people within society—meaning researchers often control the narrative around autism.

The aim of this mixed-method study was to understand how autism researchers construct autistic people and autism research, and to investigate whether greater inclusion of autistic people in research relates to lower odds of cues of ableism in narratives about autistic people. To achieve this, our study had both a quantitative and qualitative element. For the quantitative part, we used content analysis to code narrative stance used by autism researchers (medical versus social model) and for cues of ableism (dehumanization, objectification, and stigmatization). Then, we investigated whether narrative stance and degree of participatory research predicted odds of ableist cues (while controlling for career length and contact with autistic people). We predicted that alignment to the medical model would relate to a higher odds of cues of ableism about autistic people. Further, we predicted that higher participation of autistic people in the research process would relate to lower odds of the presence of such cues. For the qualitative part, we used reflexive thematic analysis to broadly and inductively examine how researchers construct autistic people and the autism research process. The qualitative analysis was used to contextualize the quantitative findings, and to add depth to the study.

## Materials and methods

### Recruitment methods and participant criteria

Ethical approval was obtained from the University of Stirling General University Ethics Panel, and all participants gave informed consent before starting the survey. We defined an autism researcher as anyone who has conducted or is conducting research about autism including those from outside of academia, and at any career stage. We aimed to recruit participants from around the world, although limited to English-speaking participants. We used multiple approaches to recruit participants. Initially, we shared a study advert on our research-related Twitter accounts in February 2021. Combined, we had almost 5,000 followers at the time, many of whom are autism researchers, although it is likely many of these researchers aligned with our own research areas, perspectives or were UK-based. As such, we also used purposive sampling to target autism researchers beyond our social media reach. To do this, we posted an advert on an autism researcher mailing list for those interested in Higher Education research, which has approximately 450 people on the list, mainly in the USA. We also developed a list of autism researchers particularly in the medical and (hard) scientific fields (e.g., genetic, biological, neuroscientific research, etc.) by reviewing abstracts submitted to the 2020 International Society for Autism Research (INSAR) conference and papers published in the last three years, using corresponding author details. We sent an email to these researchers including study information and link to the survey, and we contacted 981 researchers in this way. We recruited approximately 126 participants via Twitter or the mailing list, and 69 via targeted emails (7% email response rate). Recruitment was open between February and June 2021. We did not expect this to be a fully representative sample of the field, and instead aimed to capture as many perspectives as possible within the limitations of self-selection methods.

### Participants

Overall, 195 participants took part. Most were from Westernized countries, particularly the United Kingdom and North America, and were predominantly white (Table 1). Most identified as female (*n* = 124), with 41 male, three non-binary and two genderqueer participants (25 participants did not specify). The mean age of participants was 37.78 (*SD* = 11.90; Range = 20–75, *n* = 169). In terms of personal connections to autistic people outside of research, many reported they had autistic family members, work colleagues or friends, with almost 20% reporting they were autistic themselves (Table 1).

**TABLE 1:** Participant demographic information.

	*N*	%
**Country (*n* = 174)**		
UK	64	36.8
North America	58	33.3
Australia and New Zealand	23	13.2
Europe other	21	12.1
Asia	3	1.7
Africa	2	1.1
South America	2	1.1
Middle East	1	0.6
**Ethnicity (*n* = 170)**		
Asian	7	4.1
Hispanic/Latino	4	2.4
Middle Eastern	1	0.6
Mixed/Multi-ethnic	4	2.4
Native people/indigenous	1	0.6
Pacific Islander	1	0.6
White/Caucasian	148	87.1
Prefer not to say	2	1.2
Other	2	1.2
**Education (*n* = 170)**		
High school qualifications or equivalent	1	0.6
Undergraduate/Bachelor’s degree or equivalent	18	10.6
Masters degree or equivalent	45	26.5
Doctoral degree/MD or equivalent	106	62.4
Employment* (*n* = 173)		
Employed full-time	103	59.5
Employed part-time	28	16.2
Self-employed	4	2.3
Unemployed	2	1.2
Unable to work	2	1.2
Retired	1	0.6
Student	48	27.7
Carer	3	1.7
Personal connections to autistic people* (*n* = 173)		
I am autistic	33	19.1
I have an autistic family member or partner	73	42.2
I have autistic friends, colleagues or family friends’ children	119	68.8
I do not have any personal connections with autistic people	34	19.7

Some participants elected not to provide some demographic information thus total *n* responses for each question are reported below. *Percentages add up to more than 100% since participants could select more than one option.

Participants came from a range of career stages or roles within autism research, academic disciplines, and research areas (Table 2). However, most were from Psychology and investigating services and societal issues. The mean length of time participants had been involved in autism research for was 8.47 years (*SD* = 7.48, range: 1–40 years, median = 6 years, *n* = 172). Mean time spent on autism research across participants was 75.33% (*SD* = 26.03; range: 5–100%, median 85%, *n* = 172).

**TABLE 2:** Information regarding participant’s research backgrounds, including discipline, research areas and career stage.

	*N*	%
Academic discipline (*n* = 173)* Psychology Education Medicine-allied fields (e.g., psychiatry, pediatrics) “Hard” sciences (e.g., biology, epidemiology) Arts and humanities (e.g., sociology, disability studies)	110 51 42 40 35	63.5 29.4 24.2 23.1 20.2
Research area (*n* = 174)* Services and societal issues Treatments, interventions and causes Biology, brain and cognition Diagnosis, symptoms and behavior	102 88 70 65	58.6 50.5 40.2 37.4
**Career stage (*n* = 174)** Senior e.g., Professor or Senior lecturer Early e.g., Lecturer, Post-doctoral fellow In training e.g., Ph.D or Masters student Non-academic e.g., consultant, charity	48 51 61 14	27.5 29.3 35.1 8.0

*Participants could select more than one option.

### Materials and procedure

We used a mixed methods survey, hosted online using “Qualtrics.” Participants completed the questions in the order presented below. Participants first selected their personal language preferences for talking about autism (e.g., autistic person, person with autism, no preference) so the survey displayed their preferred terminology in subsequent questions. The next set of questions were qualitative, aiming to collect autism researchers’ spontaneous thoughts about autism, autistic people, and autism research, using five open questions. We asked participants to supply as much detail as possible in response to the following questions: (1) Describe what autism is, (2) describe autistic people, (3) describe the cause of autism, (4) describe what you think the main goal of autism research in general should be, (5) describe the main aim of your own research. We used this approach to limit the possibility of demand characteristics, as researchers might have recognized the purpose of psychometric scales for stigma or dehumanization.

The next questions recorded information such as the participant’s area of research, based on Pellicano et al. (2014) organization of autism research topics, and their academic discipline. For these questions participants could select multiple options. Additionally, we asked participants to select their current job title from a list of options, number of years working as an autism researcher, current country, and percentage of research focused on autism.

Next, participants answered questions about language usage within their research. Specifically, we asked how they most often referred to autism in their research (e.g., autism, autism spectrum disorder, autism spectrum condition), how they referred to autistic people (e.g., autistic person, person with autism, person on the autistic/autism spectrum), and how they referred to non-autistic people (e.g., typically-developing, neurotypical, people without autism, etc.). For these questions, participants could only select one option. They then answered questions about their connections with autistic people outside of research, selecting from options such as “I am autistic,” “I have an autistic family member,” “I have autistic friends.” Here, participants could select multiple options.

Based on Arnstein (1969) ladder of participation, we asked participants about their experiences of involving autistic people in their research. We presented participants with a series of options (randomized) and participants had to select all statements that applied to them. The lowest option on the ladder (rank = 1) was “I have not involved autistic people in my research” and the highest option (rank = 9) was “I am an autistic autism researcher.” For non-autistic autism researchers, the highest possible option (rank = 8) was “I have worked collaboratively with autistic autism researchers.” Further details on the ladder of participation can be seen in Table 3 in the results, including further categorization. Finally, participants could optionally answer questions regarding personal demographic information such as gender, ethnicity, and age. The survey took approximately 22 min to complete.

**TABLE 3:** Highest degree of participatory research researchers have undertaken (*N* = 170), based on Arnstein (1969) ladder of participation.

		*n*	%
Non-participatory research	I have not involved autistic people in my research	23	13.5
	It is not possible to involve autistic people in the research I do	6	3.52
Tokenistic research	*Informing:* I have shared my research findings with autistic people	113	66.5
	*Consulting:* I have asked autistic people for their views on a research project I designed	99	58.2
	*Placation:* I have surveyed autistic people to find out what research ideas they are interested in and then decided what to research based on this information	47	27.6
Participatory research	*Partnership:* I have worked in partnership with an autistic person to design a research project	55	32.4
	*Delegated power 1:* I have worked with autistic people who were not trained researchers, but we worked together to conduct research from start to finish	44	25.9
	*Delegated power 2:* I have worked collaboratively with autistic autism researchers	64	37.6
Citizen control	I am an autistic autism researcher	34	20

### Data analysis

Firstly, we used content analysis followed by logistic regression to quantitatively investigate whether positionality and degree of participatory research predicts cues of ableism (sentiments which dehumanize, objectify, or stigmatize autistic people) in narratives while controlling for years in autism research and contact with autistic people. By controlling for these variables, we can ensure that alternative explanations of the outcome variable are not better explained by potentially related variables. Secondly, we conducted a bottom-up inductive, reflexive thematic analysis to qualitatively analyze the narratives of researchers.

#### Content analysis

We used deductive content analysis to code (1) participants narratives into narrative positioning (medical/social), and (2) for cues to dehumanize, objectify, or stigmatize autistic people.

*Positioning:* For positioning (medical/social), we created a coding scheme based on existing literature. Narratives which focused on autism as a medical disorder, as situated within an individual, which stressed individual impairment or disease, we coded as “medical model.” For example: “*Impairments in social communication with stereotyped and repetitive behaviors beginning early in development. It has a highly variable manifestation, with some individuals non-verbal and lacking self-care capacity, and others able to function extremely well*.” Narratives which focused on the social or cultural barriers as the root of impairment in autism, we coded as “social model,” for example: “*Growing up autistic [*…*] attracts stigma and stress to the individual and also their family. This is because of prejudice, intolerance and lack of reasonable accommodations*.”

However, we realized that the original coding scheme was inadequate, and identified a third category of “neutral embodiment.” This third positioning placed autism within an individual but described it as a neutral difference, and typically described ways in which autistic people interacted with or processed information around them, for example: “*Autism is part of a constellation of ways of being*.” One hundred and seventy-two participants provided sufficient information that we could code into one of the three narratives. Both the social model and the neutral embodiment narratives fit within a neurodiversity approach.

*Cues for dehumanization, objectification, and stigmatization:* We used definitions within literature of each of the concepts to create the coding scheme. Each concept with its definition and exemplar quotes is available in Table 4.

**TABLE 4:** Ableism cues, their definition and example quotes.

Cue types	Definition	Exemplar quotes
Dehumanisation (Haslam, 2006)	Denial of full humanness, infantilisation, compared to non-human animals, treated as lacking in rationality, refinement, cultural inability, as passive, or fungible, or machine-like.	“autism people may demonstrate an apparent absence of empathy for people’s feelings, lack of responsiveness, awareness of social cues or cultural norms.”
Objectification (LaCroix and Pratto, 2015)	Instrumentality, denial of autonomy, inertness, violability, ownership, denial of subjectivity, or fungibility	“Without often comorbid intellectual disability, and dependent upon the severity of their autism, they are interesting, often charming, and valuable members of the community.”
Stigmatizing	Strongly stereotyping, risk-based language, strong othering based on normative expectations	“shut down from the outside world; rigid; emotional; fat”

Both researchers coded all the data independently, before calculating inter-rater reliability. Initial agreement after independent coding ranged from 68 to 85% for each positioning, and 63% for stigma cues. Lower agreement for stigma was potentially because of a lack of detail in the coding scheme, which was amended to be more detailed to make decision-making less ambiguous. After discussion, we recoded and reached 85–92% agreement for positioning and 84% for stigma cues (indicating that adding detail was successful for increasing agreement). We then met again to discuss all disagreements, until we reached 100% agreement. For accounts where we could not agree (*n* = 3 for positionality), an external individual independent of the project provided their views, and we reached agreement. The external evaluator was also an autism researcher but otherwise uninvolved in the project.

To analyze predictors of stigma cues, we conducted hierarchical logistic regression. The outcome variable was the presence of ableist cues (binary variable: present or not present). Model one included years in autism research as a predictor. Model two added familial and non-familial contact with autistic people. Model three (the final model) added positioning and degree of participatory research (as a linear scale from non-participation to citizen control).

#### Reflexive thematic analysis

Following the quantitative element, we used thematic analysis (Braun and Clarke, 2006). This involved each author analyzing half of the scripts each (split according to odd/even numbers). Firstly, we refamiliarized ourselves with the qualitative data and then proceeded to do semantic and latent coding to create initial codes. We then met to discuss potential themes that we were generating. We had high agreement over the themes and reviewed all responses with those themes in mind. Finally, we named the themes, generating names until they were clear and best represented the underlying data.

### Epistemic stance and reflexivity

Critical realism presupposes a singular objective reality which exists independent of, and often prior to interaction with it, but also that any and all description of reality is mediated through language, social cultural context, and meaning-making (Oliver, 2012). This makes the stance ontologically realist, but epistemically relativist (Bhaskar, 1997). Acknowledging that regardless of the ontology of autism, all knowledge *about* autism is situated, we took a reflexive stance to aid recognition and transparency in how our own positions shaped our interaction and construction of the data. Thus, we both used reflective memos and note-taking throughout the research process, as well as regular de-briefing, member-checking, and discussion of our own positionalities and values. One author is autistic while the other is non-autistic, and we both acknowledge we have different proximities and positions in relation to the data. A reflection is provided in the discussion. This is also important in line with critical realism, which acknowledges that while there is an independent singular reality, all representations of it (including knowledge made in research processes) are mediated through experiences and language.

## Results

### Quantitative analysis

*Descriptive statistics for language preference and autistic participation in research* Participants’ language preferences, both personally and professionally within their research, are presented in Table 5. Interestingly, there was a difference between the language researchers preferred and what they used in their research.

**TABLE 5:** Language preferences of researchers when referring to autism and autistic people.

	*N*	%
**Personal language preference (*n* = 195)**		
Identity first (autistic person)	110	56.4
Person first (person with autism)	27	13.8
No preference	58	29.7
**Language most often used in research when referring to autistic people (*n* = 172)**		
Identity first (autistic person)	103	59.9
Person first (person with autism)	48	27.9
Person on the autistic/autism spectrum	13	7.6
Other	8	4.7
**Language most often used in research when referring to autism (*n* = 172)**		
Autism spectrum disorder (ASD)	63	36.6
Autism spectrum condition (ASC)	12	7.0
Autism	91	52.9
Other	6	3.5
**Language used most often in research when referring to non-autistic people**		
Typically-developing/typical	45	26.2
Neurotypical, allistic or other neurodiversity-related terms	58	33.7
Healthy controls/controls/normative	4	2.3
Non-autistic	49	28.5
People/individuals without autism	13	7.6
Other	3	1.7

For the ladder of participation, participants could select multiple options concerning their approaches. Table 3 shows the individual options selected by participants. We then categorized participants (*n* = 170) at their highest selected option on the ladder of participation as either conducting non-participatory (*n* = 21, 12.4%), tokenistic (*n* = 40, 23.5%), participatory research (*n* = 75, 44.1%) or as an autistic autism researcher (*n* = 34, 20.0%).

We coded most researchers as having a medical model narrative (*n* = 96, 55.8%), followed by neutral embodiment (*n* = 56, 32.6%) and social model (*n* = 20, 11.6%). For stigma cues, we coded these as present in 104 participants (60.1%).

### Predictors of cues of ableism in narratives

We used hierarchical binary logistic regression to investigate whether narrative positioning and degree of participatory research involvement significantly predicted the odds of ableism cues while controlling for years in autism research and connections with autistic people (Table 6). By controlling for these variables, we can ensure that alternative explanations of the outcome variable are not better explained by potentially related variables. First, to ensure the data met assumptions of logistic regression we checked for outliers using Mahalanobis distances, and there were no significant outliers in continuous variables. Second, we checked for multicollinearity by checking correlations between predictor variables to ensure no variables were highly correlated, and then through variance inflation factors (VIF) and condition indices. A significant high correlation (*r* > 0.80) may signal multicollinearity, but our highest correlation was substantially lower (*r* = 0.36, *p* < 0.001). We used a conservative cut-off of 3 to check for multicollinearity, and VIF were below this limit (VIFs ≤ 1.25). While a high condition index (15–30) signals multicollinearity, the largest condition index was below this (<10). Lastly, we used the Box-Tidwell test to check for linearity between the predictors and the logit for continuous variables. These were non-significant (*p* > 0.05) suggesting the assumption of linearity between predictors and the logit was met.

**TABLE 6:** Binary logistic regression results predicting cues of ableism (present or not present).

	*B*	SE	Wald	df	*P*	Exp (B)	95% CI	
**Model one**								
Years in autism research	0.087	0.030	8.27	1	0.004	1.09	1.028	1.158
Constant	–0.234	0.266	0.775	1	0.379	0.791		
**Model two**								
Years in autism research	0.092	0.031	9.055	1	0.003	1.096	1.033	1.164
Familial connections	–0.036	0.343	0.011	1	0.917	0.965	0.493	1.889
Non-familial connections	–0.986	0.389	6.435	1	0.011	0.373	0.174	0.799
Constant	–0.060	0.282	0.045	1	0.833	0.942		
Fit compared to model one	X^2^ (2) = 7.29, *p* = 0.026							
**Model three**								
Years in autism research	0.059	0.035	2.89	1	0.089	1.06	0.991	1.14
Familial connections	0.643	0.437	2.17	1	0.141	1.90	0.808	4.48
Non-familial connections	–0.665	0.471	1.99	1	0.158	0.514	0.204	1.29
Narrative (medical)			26.4	2	< 0.001			
Social model	–1.82	0.448	16.5	1	< 0.001	0.162	0.067	0.390
Neutral embodiment	–2.89	0.676	18.28	1	< 0.001	0.056	0.015	0.210
Participatory research	–0.504	0.243	4.23	1	0.038	0.604	0.375	0.973
Constant	1.03	0.787	1.72	1	0.190	2.81		
Fit compared to model two	X^2^ (3) = 45.8, *p* < 0.001							

#### Model one

In model one, we included the control variable “years in autism research” as predictor of stigma cues. Years in autism research significantly and positively predicted a higher odds of ableism cues (*p* = 0.004).

#### Model two

In model two, we added the next two control variables (familial connections and non-familial connections to autistic people). Years in autism research remained significantly and positively predictive of higher odds of ableist cues (*p* = 0.003). Familial connections to autistic people was non-significant (*p* > 0.05). Non-familial connections significantly predicted lower odds of ableism cues (*p* = 0.01).

#### Model three

Model three retained the control variables as above, and added “Narrative” (medical narrative was the comparison category) and “Degree of participatory research.” No control variables were significant in model three (*p* > 0.05). Narrative positioning significantly predicted ableism cues, such that the medical model predicted higher odds compared to both the social (*p* < 0.001) and neutral embodied narratives (*p* < 0.001). Additionally, greater participatory research predicted lower odds of ableism cues (*p* = 0.03). Full statistical results for each model are available in Table 6.

### Thematic analysis

**FIGURE 1:**
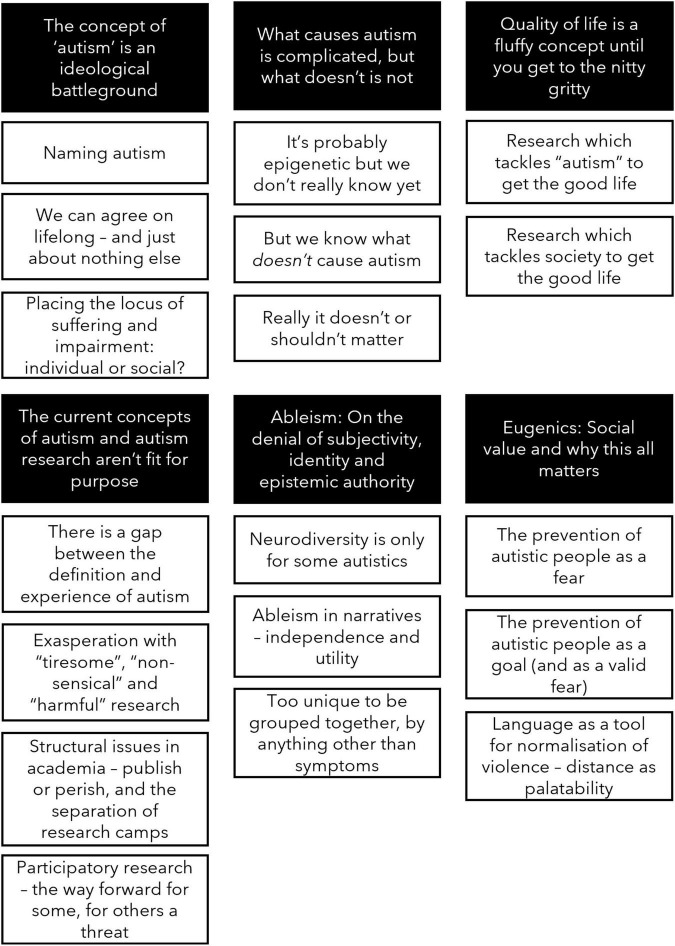
Thematic map showing the themes and sub-themes identified.

We identified six themes which are outlined below, including subthemes. These themes are mapped visually in Figure 1.

#### “The concept of “autism” is an ideological battleground”

This theme reflected how participants often seemed to struggle with the concept of “autism,” with a range of disagreements and contradictions between (and even within) researchers. We identified several sub-themes which highlighted the core issues:

##### Sub-theme: Naming autism

Participants’ descriptions of autistic people, and autism more broadly, were varied and reflected the core disagreements about the ontology of autism. For example, autism was described as a “disease,” “disorder,” “condition,” “difference,” “neurodivergence,” “minority neurotype” and a “disability.” Although language varied, many relied on medicalized notions of autistic people which reflected the DSM-5 as mostly as “*a neurodevelopmental disorder*” (e.g., ID99) but also as “*a disease which varies widely*” (ID100). For example:

*“Autism Spectrum Disorder represents a neurodevelopmental disorder characterized by deficits in social communication, social interaction and obsessive/stereotyped patterns of behavior, interests or activities”* (ID101).

However, this was not the only way participants had defined autism, with some describing instead “*autism is a spectrum of difference where one could be described as neurodivergent*” (ID102), as “*a neurotype that is not the majority*” (ID103) or as “*a neurodevelopmental disability which includes a spectrum of abilities and challenges*” (ID104).

##### Sub-theme: We can agree on lifelong—and just about nothing else

There was a general agreement across researchers (regardless of how they constructed autism) that autism is lifelong—“*Autism is a lifelong neurodevelopmental condition*,” (ID105), “*people are born autistic, and remain autistic throughout their lives*” (ID106), “*Autism is a neurodevelopmental disorder, and is a lifelong diagnosis*” (ID107).

##### Sub-theme: “The good life”—placing the locus of suffering and impairment: Individual or social?

There was substantial variation in where researchers placed the locus of impairment or suffering with regards to autism, and it appeared to relate to their constructions of autistic people. Where researchers had defined autism as being a disorder, disease or condition, they tended to locate suffering and impairment as internal to autistic people, but ascribed to an amorphous “autism,” which radiated outward to disrupt the lives of those around the person, for example: *“Autism*… *may severely impair the life of the affected subject, as well as that of her/his relatives*” (ID108) and:

*“While a diagnosis of autism is by no means a “death sentence,” nor does it “doom” the individual to any specific life, it does present a wide variety of challenges to families who may not be prepared to help their loved one with the diagnosis*” (ID109).

Alternatively, researchers who constructed autism as a difference, disability or as a minority neurotype, tended to still place autism within an individual (as a biological or genetic phenomenon, but not treating autism as amorphous), and placed most suffering or impairment externally in society or the environment, for example:

“*It is the society we live in, the lack of support available and the ableist and heavily gendered expectations that exist which affect autistic people on a daily basis and lead to autism being described so negatively, even by other researchers”* (ID110).

Indeed, some pointed to a chronic lack of accommodation made for autistic people: “*Many autistic people experience depression and/or anxiety; this is not inherently part of being autistic but may be associated with living in a world not designed for you*.” (ID111).

#### What causes autism is complicated, but what doesn’t is not

We directly asked participants what they thought caused autism. In their answers, there was a pattern of participants purporting that the causes were complicated, although there were some that could be ruled out. These points are explored further within our subthemes:

##### Sub-theme: It’s probably epigenetic but we don’t really know yet…

Researchers predominantly ascribed the cause of autism to an interaction between genetics and environment, while similarly hedging their bets because it is both complicated and not yet fully known. Participants expressed uncertainty with phrases like “*this is complicated*” (ID112) “*it’s not clear*” (ID113), “*not known with certainty*” (ID114) and “*there is not consensus*” (ID115). Despite this, a majority settled on some form of gene-environment interaction:

“*There is likely a genetic component, though numerous genes are implicated. There is also evidence that certain environmental variables influence risk of later diagnosis. The answer is likely a combination of genetic and environmental variables*” (ID115).

Less prevalent were participants who ascribed more of the cause of autism to social and cultural processes, as well as biological ones:

*“Many different cascading effects cause autism: from genetics to brain chemistry to societal norms to random chance. It can’t be pinpointed to one particular thing*…” (ID106).

##### Sub-theme: But we know what doesn’t cause autism

Participants were more certain around what does *not* cause autism, for example, “*Definitely not bad parenting!”* (ID116) and “*NOT related to vaccinations which is a common myth*” (ID117). One participant noted:

“There is no known cause of autism, despite a huge amount of research funding and time being invested in this question. It’s definitely easier to answer the question of what *doesn’t* cause autism: vaccinations and poor parenting being particularly notable” (ID106).

Dispelling these notions seemed to be important to people specifically because of the damaging notion which placed the fault with parents:

*“It was theorized at one point that autism was caused by “cold parenting” but this was not based on any evidence and was extremely damaging to families at the time”* (ID118).

##### Sub-theme: Really it doesn’t or shouldn’t matter

Some respondents were “*not terribly interested in the cause and beyond being confident that it is not due to refrigerator mother theory*” (ID111), since it would not change the everyday existence of autistic people or improve their lives. Another participant elaborated:

*“I have no interest in understanding cause. I want to know how we can best support autistic people in ensuring equitable access to resources, community services, employment, etc, and helping to turn the conversation to changes the non-autistic community must make to facilitate this support, understanding, inclusion and respect”* (ID119).

Others pointed to the fact that autistic people simply exist because humanity does: *“Humanity is the cause of autism. Humans are diverse, by definition. The very nature of diversity in humanity yielded the neurodiversity of autism”* (ID120).

#### Quality of life is a fluffy concept until you get to the nitty-gritty

Overall researchers appeared to share similar goals at a high, abstracted level—there was a focus on how research should improve quality of life for autistic people. However, “quality of life” is a nebulous term, until participants detailed what they considered to be barriers of quality of life for autistic people—some found poor quality of life to be inherent to “autism,” while others consider it to be determined by society, as explored in our subthemes:

##### Sub-theme: Research which tackles “autism” to get the good life

Participants who tended to construct autism as a disease, disorder or condition and typically describing “autism” as an amorphous concept saw tackling autism as the goal of autism research. Here, autism was a barrier to a good life:

*“[The goal of autism research should be] to improve the life of autistic people, to allow them to achieve everything that they wish to without their disorder getting in the way”* (ID100).

For many, the focus was on remediation, treatment, and ultimately the prevention of “autism”: “*The ultimate goal is to develop novel therapies against autism*” (ID121). This goal included eugenic prevention of autistic people, and how this might provide benefits for others:

*“Autism research should pursue multiple goals [including] understanding the genetics of autism-associated neurodevelopment. This may ultimately lead to screening, genetic counseling of parents, and potentially, prevention*… *Researching the collateral benefits of treatment for individuals with autism and their families and other relevant persons*” (ID122).

##### Sub-theme: Research with tackles society to get to the good life

In contrast, those who constructed autistic people as being disabled, neurodivergent or a minority neurotype, and often referring to autistic people rather than amorphous “autism,” described how autism research should focus on society, culture or environments as the cause of disablement and suffering in autistic people’s lives, for example:

*“Research should look into ways to address inequalities affecting autistic people, such as increasing inclusion and acceptance in society, and support wellbeing, quality of life, independence and autonomy”* (ID123).

Instead of tackling “autism,” there was a focus on tackling systems which are imbued with neuro-normativity, to facilitate access to the world for people who think about and experience the world differently:

*“I think that the main goal of autism research should be to improve the lives on autistic people by making the world an easier place for them to exist in. So much of how our society works is based on normative behaviors when high percentages of the population do not think in a typical way. Autism research should also consider, include, and center the voices and the wishes of autistic people”* (ID124).

#### The current concepts of autism and autism research aren’t fit for purpose

This theme encapsulated the view that how we currently conceptualize “autism” and how we do autism research is beset with issues. These issues are explored further in our subthemes:

##### Sub-theme: There is a gap between the definition and experience of autism

Regardless of participants stances, there was a dissatisfaction with the definition of autism and autism research, with participants highlighting the distinct difference between how autism is defined, versus how it is experienced:

*“The official “definition” of autism in the States per the DSM is a neurological disorder characterized by repetitive behavior and difficulties in social communication. This, of course, is distinct from the lived experiences of people with autism and their families and friends”* (ID125).

Some participants acknowledged that while the DSM was important, the criteria had clear weaknesses:

*“I believe that while the DSM-5 criteria are important, there are fundamental flaws in that they do not account for the nuanced variations in the ways in which autism presents*” (ID126).

Participants highlighted that this was particularly the case for autistic people at intersections of marginalization—given current definitions of autism center around white, cis-gender boys:

“*The criteria used to diagnose autistic people is outdated and, in my view, discredited. It is over reliant on stereotypes now discredited and barely considers female autistic traits”* (ID127).

*“More males are diagnosed than females, though this may be due to biases in the diagnostic criteria and the tests developed to diagnose autism. People of color are more likely to be diagnosed later than white people, also likely due to biases in the diagnostic criteria and racism”* (ID111).

##### Sub-theme: Exasperation with “tiresome,” “non-sensical,” and “harmful” research

Some participants expressed an exasperation with autism research as a process—for some it was outdated: “*Autism research has been very outdated for a number of years, utilizing tired ideas such as the extreme male brain theory*” (ID127), and others went as far as to question the point:

*“Increasingly, I’m not convinced that there is a good case for autism research to continue. I have now spent 10* + *years in autism research, and the longer I stick around, the less I am convinced that autism is different or special from other parts of the human experience, and other issues related to equality, diversity, and minority communities”* (ID128).

Part of this dissatisfaction was with the amount of funding which went to biomedical research, with a participant saying: “*I find the current and historical fascination with the biogenetic causes of autism tiresome*” (ID106). Another participant explained:

*“At present much money is wasted on biomedical research projects that are naive, impractical, and/or pointless*…*For example, people waste time trying to find neurobiological markers of autism, but what is the point of that? You still need to find someone to discuss [diagnosis] with family/individual (hopefully someone more capable of critical thought than the people who say all the value-laden, subjective, often offensive clinical discourse). So I would like to see less of the neurobiological research, and for the remainder of that research to be subjected to additional critical scrutiny”* (ID129).

This was a shared concern with others feeling that money spent on causes or genetics was a waste:

*“I think autism research should stop in attempting to identify the “cause” or “gene” of autism. It wastes valuable money and we have spent over 20* + *years and billions of dollars with outcomes showing there is no single gene and no single cause. we need to move on”* (ID130).

Participants highlighted that research often set autistic people up to fail, or was shaped in such a way that resulted in autistic people always being viewed as deficient:

*“We know so much (though really, still so little) about non-autistic cognition, interaction, and perception. But comparatively little about autistic people and what research we do have is often from the perspective that autistic people are “worse” at whatever it is than non-autistic people. More work needs to be done to develop tests and measures that aren’t predisposed to “fail” autistic people”* (ID111).

##### Sub-theme: Structural issues in academia—publish or perish, and the separation of research camps

There were concerns that research had become a paper mill devoid of any value for autistic people, and that researchers focused on their own careers in a publish-or-perish mentality:

*“It [research goal] shouldn’t be publishing papers, which seems to be its main goal at the moment. It should be collaborating with autistic individuals to actually have an impact in areas that are important to them. Great you published another paper but how does that impact an autistic child in local primary school”* (ID131).

Further, that change was made impossible by structures in academia that resulted in siloed knowledge bases, preventing bridges between disciplines to fully appreciate autistic people. Participants pointed to the fact that contrary knowledge was not necessarily seen as valid or relevant:

*“My concept of autism has grown to include the theory of double empathy and much of the current published work related to that, but as my research is in genetics and biomarkers of autism, most peer reviewers in the field have never heard of the concept and even so would probably not find it relevant, compared to peer reviewers in social sciences.”* (ID132).

##### Sub-theme: Participatory research—the way forward for some, for others a threat

There were mixed views about autistic people’s involvement in research, and there were polarized views on what increased involvement meant. For some, the involvement of autistic people in research represented hope, expressing that it is “*encouraging to see that there are more autistic people being actively involved in research*” (ID118). This was a shared hope that “*research is thankfully progressing*”(ID127):

*“I am hugely encouraged by the rise of autistic researchers and by co-produced research doing research that could benefit autistic people rather than see them as subjects to do research to”* (ID133).

For some, autistic involvement was key and represented moving on from either harmful or pointless research:

*“Research should be what autistic people want it to be about. Not a cure, not mice experiments, nor trying to explain yet more reasons for us to be deficits. Research how we can be actually accepted into society, how things can improve our wellbeing, how the tired and harmful language of historical bias in literature can be put right for the whole of society to change their attitudes they have based on the rhetoric of medicalized deficit disablement of papers. Put right what has been wronged”* (ID134).

Yet for others, autistic people being included in autism research, or challenging what they perceived as harmful or outdated research, was seen as a threat which jeopardized non-autistic peoples place in making autism research:

*“Autism research is in crisis. Non-autistic people/researchers who have dedicated decades to studying autism are being pushed out of the field”* (ID135).

#### Ableism: On the denial of subjectivity, identity, and epistemic authority

This theme captured some of the ways in which participants talked about autistic people in ways which were dehumanizing, objectifying, and stigmatizing. These aspects are elaborated on within our three subthemes:

##### Sub-theme: Neurodiversity is only for some autistics

Perspectives which placed disablement in society or focused on social and cultural roles in producing outcomes for autistic people were described as rose-tinted, only applicable to what were often described as “high-functioning” autistic people, or as a less “scientific” or anti-scientific approach. The idea of having higher-support needs, an intellectual disability, or being more obviously different seemed to undermine claims of neurodiversity for some who placed a high value on normative functioning:

*“Some refer to autism as a condition or neurodiversity. I think that it really depends on where along the spectrum (which includes multiple dimensions) one might be”* (ID99).

Participants highlighted non-speaking autistic people, and autistic people with intellectual disability, as group for which neurodiversity or autistic rights movements could not necessarily be helpful for:

*“As much as I encourage the autism advocacy movement, there is a deafening silence from a large proportion of autistic people, namely those with an intellectual disability/limited communication”* (ID136).

Others argued the idea of autism as a social, political, or community descriptor as “under-evidenced,” but did not clarify what evidence would be sufficient:

*“Some persons also claim autism as a descriptor of a social, political, or community identity, and make further claims about uniquely autistic styles of interaction or communication. As yet, there has been little research done that would support or refute those claims”* (ID128).

##### Sub-theme: Ableism in narratives—independence and utility

Ableism was pervasive in narratives about autistic people. Some of the dehumanization of autistic people was clear, whereby participants described autistic people as being unresponsive, culturally incapable, rigid, and lacking in complex emotions: *“Autism people [sic] may demonstrate an apparent absence of empathy for people’s feelings, lack of responsiveness, awareness of social cues or cultural norms.”* (ID137). When asked to describe autistic people, one researcher said: “*shut down from the outside world; rigid; emotional; fat*” (ID138).

Similarly, some participants described autistic people as being almost irrationally emotional or completely emotionless, alongside being incapable of more complex secondary emotions:

*“People with autism are very heterogeneous. They can speak, write, as well as smile and hug or to be completely inexpressive and apparently without emotions [*…*] In the absence of intellectual disability they can perform many activities*…” (ID139).

The utility of autistic people (which appeared to hinge on perceived intellectual ability and independence) determined the perceived social value of autistic people. Where strengths were highlighted, they were often framed in terms of the utility of “*high-functioning autistic people*… *[who are] extremely gifted persons with high but restricted professional skills (e.g., in data classification/analysis)*” (ID121), or *“Common characteristics such as attention to detail, intense interests and honesty can be an asset in many professions”* (ID140).

As one participant noted, “*autistic people [are] only seen as of being useful or valuable if they contribute to the wider economy*…” (ID127). This is an example of objectification because it demonstrates that autistic people do not have worth unless they produce value for other people. While some participants focused on “*interventions that help individuals with autism live normal, happy and productive lives*” (ID147) (our emphasis), others appeared to rule out value based on the presence of any intellectual disability:

*“Without often comorbid intellectual disability, and dependent upon the severity of their autism, they are interesting, often charming, and valuable members of the community”* (ID141).

##### Sub-theme: Too unique to be grouped together…by anything other than symptoms

Autistic people were denied identity and community as linking them, and when asked to describe autistic people, many argued that it was “*impossible*” (ID148), and yet, went on to group autistic people by a list of symptoms. Further, those who referred to autism as an amorphous object often attributed heterogeneity to autism:

*“As autism is so heterogenous this is a difficult question to answer. Medically speaking, autism people have difficulty with social communication and interaction. For example, eye contact may be limited, and “rules” of interactions can be challenging, e.g., turn taking, ensuring everyone is interested in the topic, staying on topic”* (ID140).

This was distinct to how others positioned autistic people as heterogenous because of the heterogenous nature of humanity, or because of other identities autistic people may hold:

*“Autistic people are as diverse as non-autistic people, both demographically and in terms of their personalities, interests, views, and experiences”*(ID142).

Others argued that autistic people were *so unique* that they lacked commonality with each other more so than non-autistic people:

*“Autistic people are a heterogenous group, many of whom share similar experiences related to the myriad combinations and degrees of symptoms I described in the previous question. There is likely as much or more difference among autistic people than between autistic and non-autistic people”* (ID143).

It appeared that some participants wanted to stress the humanness of autistic people to prevent or counteract dehumanization or objectification, but in doing so, erased any potential commonality of identity, community, shared experience, or collectivism, which is itself a form of dehumanization. This stood in contrast to others who refused to connect autistic people through symptoms and instead grouped autistic people through the position they are given in society:

*“A minoritized group of people who are part of a particular neurotype and thus sometimes approach situations differently than those of other neurotypes”* (ID103).

Definitions like this did not constrain people to experiencing or expressing autisticness in the same way, but found commonality to unite autistic people or recognize them as a socially coherent group of people who meaningfully and challenge their position in society:

*“To create descriptions that seek to find common identities within this group should be looking outward to the common stigma and oppression that society too often inflicts”* (ID144).

#### Eugenics: Social value and why this all matters

The final theme brought together some of the most concerning patterns in the responses regarding eugenics and the eradication of autistic people. This theme is expanded on within three subthemes:

##### Sub-theme: The prevention of autistic people as a fear

There was a repeated underlying concern from many participants that eugenic traditions were still present in the goals of research: “*cause-focused research also makes me nervous in terms of the potential for eugenics*” (ID111). These concerns pervaded some researchers’ discourse, believing the ambiguity of the cause of autism (as discussed earlier) was the only barrier standing between autistic people and the prevention of autistic people:

*“If the cause of autism was known, I fervently believe this would be a terrible thing. Eugenics and social engineering, consciously and subconsciously, would take place to I believe prevent autistic people from being born, or at the very least ensure they were born with non-autistic traits”*(ID111).

Participants viewed the focus of preventing autistic people as deeply problematic:

*“Research should focus on helping autistic people already here, rather than eugenic solutions which seek to prevent and generate fear around hypothetical future autistic people”* (ID143).

##### Sub-theme: The prevention of autistic people as a goal (and as a valid fear)

Although some painted autistic people as being anti-science for expressing concerns around the genetic removal of autistic people from society, this was an active goal of other researchers, who constructed it as what the primary goal of autism research should be:

*“[The goal of autism research is] to pinpoint genetic and non-genetic etiologies [sic], disease modifiers, create awareness, strategies for early screening and diagnosis, develop new or improved interventions or preventions”* (ID145).

This included intervening before or during pregnancy and through genetic counseling:

*“I think the main goal should be to understand what causes autism and what also impacts the trajectory of autism for individuals over their lives. By understanding the former we may be able to intervene for parents before they have a child*…” (ID113).

##### Sub-theme: Language as a tool for normalization of violence—distance as palatability

This sub-theme related to the amorphous “autism” discussed earlier, whereby when talking about the prevention or eradication of autistic people, participants only referred to the prevention or eradication of “autism” and not autistic people:

*“The main goal of autism research is to, simply, learn more about it. There are scientists that research the cause (i.e.*, —*epidemiologists, etc.) and others that research how to treat it and live with it. The main goal is to learn more about*—*the why it happened and how we can help those with autism*” (ID146).

There was a focus on “preventing autism,” and never “preventing autistic people” even though, autism does not, and cannot exist as an amorphous object—autism is only ever autistic people. This was consistent with participants placing autism as within an individual and constructing it as causing devastation to those around the individual. The goal of preventing autism in this way is only possible when constructed as something that is individual, removable, separate, and inherently causing a bad life.

## Discussion

We aimed to understand and evaluate autism researchers’ narratives and language use in describing autistic people and their views on autism research. In our sample, most participants held a medical model narrative, with fewer subscribing to a social model position, although around a third held a more neutral perspective which defined autistic as a way of being. We also identified that around 60% expressed cues of ableism which we conceptualized as dehumanization, objectification, and stigmatization. In terms of what predicted these ableist cues, social model and neutral-embodiment narratives and a higher degree of participatory research predicted significantly lower odds of expressing cues of ableism, above and beyond length of research career, and familial and non-familial contact with autistic people. We used reflexive thematic analysis to further examine the narratives and views being held, and identified six themes which captured an overall sense of disparity and disagreement amongst autism researchers. This analysis highlighted not only the potential need for conceptual reconsideration of “autism” but the fundamental need for change in the way in which autism researchers talk about autistic people and do autism research.

### The future of research: Uncertainty, change and conflict

A deep frustration with the *status quo* of autism research pervaded the data, with participants unsatisfied with outdated autism research, how slowly it moves, and some questioning the relevance of autism research at all. Further, participants made clear that biases in research bled into diagnostic processes, meaning that neither the diagnostic process (including the DSM-5) nor research were fit for purpose. They highlighted the continued failure of research or diagnostic manuals to appreciate the complexity or variation of autistic people across genders or race. For example, evidence shows that autistic women and gender minorities fall through diagnostic gaps (Loomes et al., 2017). In a systemic review of 1,013 autism intervention papers across 28 years, only 25% of the articles provided data on participants’ ethnicities or race—and in studies which did report this data, white participants were included at over eight times the rate of Black participants, at ten times the rate of Asian participants, and roughly 7 times the rate of Hispanic/Latino autistic people (Steinbrenner et al., 2022). Thus, it appears to be a valid concern that autism research does not represent the experiences of many autistic people.

Interestingly, there was a gap between what participants described as their personally preferred language and the language they used in their research, whereby participants used person-first language in research, even though their preference was for identity-first. These disparities are reflected in prior debate regarding the use of person-first or identity-first language (e.g., Vivanti, 2020; Botha et al., 2021). Further, some participants in biological fields had knowledge relating to neurodiversity (such as double empathy) but felt it would not have a place in their work because peer-reviewers would not recognize this work as valid. This not only points to a fractured field, but it points to maintaining the *status quo* because of dogmatic positions in which researchers are entrenched.

There were also mixed views around what the future of autism research should look like. To participants who adopted medicalized constructions, the future still belonged to biomedical research, which consisted of treatment of current autistic people, remediation of the “core symptoms,” and prevention of future autistic people being born. However, many did report alternative accounts and directly called for a move away from cause-focused or genetic research. This approach may be more aligned with the neurodiversity paradigm and supports recent calls to move away from the medical model (Pellicano and den Houting, 2022). Pellicano and den Houting (2022) argue that the medical model takes too much of a narrow, individualistic, and deficit-focused approach, and discuss how this messaging could contribute to prejudice toward autistic people. Our data supports this point as we found a relationship between the medical model perspective and ableist cues. The medical model approach relies on the pathologization of disabled people and sustains a powerful industry built on being able to identify “deficits” and finding ways of “fixing” or “treating” these perceived deviations from an assumed norm. Since ableism is about *“a system that places value on people’s bodies and minds based on societally constructed ideas of normality, intelligence, excellence, desirability and productivity”* (Lewis, 2021), it makes sense that the medical model narrative predicted ableism cues. The future of autism research, then, may be one where we move away from the medical model.

### Participatory research and the inclusion of autistic people in autism science

Including autistic people in autism research via increased engagement in participatory research predicted significantly lower odds of ableism cues. While researchers have discussed the need for inclusion of autistic people in autism research (Chown et al., 2017; Fletcher-Watson et al., 2019, 2021), this adds to a growing body of evidence which shows that involving autistic people in autism science can be beneficial (Pellicano et al., 2021), although more work may be needed to increase researchers’ confidence and challenge systemic issues prohibiting participatory research (Pickard et al., 2022).

We also found that increased participatory research with autistic people predicted lower odds of ableism cues, above and beyond length of time in autism research or contact with autistic people (both familial and non-familial). This is important to note, because it may point to the conditions necessary for enhanced intergroup relations and for changing the ways that autism researchers discuss autism. Dynamics (such as power and agency) in familial versus non-familial scenarios may be important to understand, given that relationships between parent and child (for example) are different to *choosing* to socialize or work professionally with autistic people. Non-familial connections (which includes having autistic people as colleagues) to autistic people predicted significantly lower odds of cues of ableism, but only until we accounted for increased participatory contact, whereas familial connections did not. This highlights two key things: non-familial connections are distinct in their ability to shape the perspectives of researchers, and that informal contact with autistic people is not a replacement for actual participatory ways of working with autistic people if the goal is to develop a more humanizing autism science. However, our study cannot determine the direction of this relationship. It may be the case that those less likely to hold ableist narratives might be more likely to involve autistic people in research in the first place, or involving autistic people in research might challenge ableist narratives researchers hold. Alternatively, it may be a reciprocal relationship whereby initial openness to involving autistic people in research is required, and this involvement further changes researcher’s perceptions.

However, there is an important caveat to stress. Our quantitative analyses looked only at the utility of involving autistic people in autism science as a way of producing more humanizing autism research. Autistic people should be considered beyond this utility. As shown in the qualitative responses, autistic people are often reduced to their utility to society with little consideration given to the role society has in producing autistic people’s outcomes. Involving autistic people in autism science should warrant an understanding of what is required for equitable and non-damaging involvement. We found that researchers often dehumanized autistic people, aimed to prevent, cure, and remediate autistic people, and often othered them. We therefore need to acknowledge that when autistic people get involved in autism research, they are exposed to additional stress burden and systemic ableism (Botha, 2021). While researchers should seek and value autistic input, and this approach may shift the narrative toward affirmative accounts, systems must exist to protect autistic people involved in autism research. To date, there has been little discussion of what is required to make research hospitable to autistic involvement, how we can prevent tokenism, or ensure autistic people have equitable experiences or power in creating autism research. Although future work *with* autistic people (both researchers and non-researchers) is needed to identify exactly how we can achieve this, we would recommend systems of mentorship and support for autistic autism researchers, training for non-autistic autism researchers on avoiding ableism (see Bottema-Beutel et al., 2021) and the creation of collaborative communities.

### Ableism and eugenics: Dehumanization, objectification, and social value

The topic of ableism is one that requires further discussion, since we found ableism cues in around 60% of accounts. In our data, these ableist cues represented a denial of subjectivity, identity, culture or community, to associating autistic people with a lack of responsiveness, rigidity, through machine-like comparisons, and as both overly emotional, and at the same time, incapable of complex secondary emotions. Previous empirical research has identified that autistic people are more likely to be dehumanized by non-autistic people (Cage et al., 2018) and first-hand accounts of autism research including autoethnographies (Botha, 2021), blogs (Luterman, 2019; Rose, 2020), commentaries (Michael, 2021), and editorials (Gernsbacher, 2007; Cowen, 2009) have described that autism research is often dehumanizing. Autism research’s problem with ableism (Bottema-Beutel et al., 2020) relates to the semantic dehumanization (Haslam, 2006), objectification (LaCroix and Pratto, 2015), and stigmatization of autistic people. While some participants attempted to stress the uniqueness of autistic people, complete individuation itself separates any likeness between autistic people or shared humanity.

Further, the value and worth of autistic people often hinged on splitting autistic people into those who were seen as valuable members of society and those who were not. Most often, this utility was based upon perceived intellect and independence—for example some participants described “high-functioning” autistic people as having utility to specific professions. This point is particularly important because it demonstrates we have not moved far from the eugenic tradition of separating out autistic people into those worthy of consideration and those not (Czech, 2018). This is captured by the fact that in discussing the erasure of autistic people through pre-natal testing and intervention it is the loss of specific and valued “talent” which is lamented (Baron-Cohen, 2009). This also plays into the idea of Aspie Supremacy, in which to prove their value, autistic people with lower support needs stress the ways in which they are not similar to autistic people who have higher support needs, or are non-speaking or have an intellectual disability (de Hooge, 2019). This is even more important given that autistic peoples intelligence is often underestimated by the tools used to measure it (Nader et al., 2014).

These points also relate to the idea of neurodiversity-lite, whereby neurodiversity is adopted but only for a certain “kind” of autistic person (den Houting, 2019; Chapman, 2021). The adoption of identity-first language and support for neurodiversity only extends as far as autistic people who can make specific kinds of contributions to society, often relegating non-speaking autistic people and people with higher support needs or intellectual disabilities to the side-lines. Researchers rely on the language of neurodiversity, without enacting its core principles about the inalienable value of *all* kinds of minds and lives.

Dehumanization can also be seen in the value placed on autistic lives. The prevention of autistic people was a key concern that pervaded some participants accounts of autism research. Some described the lack of a firm cause of autism as the only thing standing between autistic people and the prevention of future autistic people. Although recent events (such as the launch of the Spectrum 10k project) saw increased debates on whether researchers are attempting to eradicate, cure or prevent autism—autistic people were said to be anti-science, or paranoid for thinking this might occur (Chapman et al., 2021). Yet, it was a clear goal for some participants. Understanding the dehumanization of autistic people by autism researchers must be understood and intervened with, because increased dehumanization of groups facilitates the permissibility of harm against them more broadly (Haslam, 2006; Moradi, 2013; Haslam and Loughnan, 2014). Further, researchers hold authority over the narrative of autism, meaning that accounts of autism which are sanctioned within academia may become ways of constructing autistic people within greater society. Autistic people are more likely to experience (poly)victimization (Weiss and Fardella, 2018), discrimination (Griffiths et al., 2019; Botha and Frost, 2020), and researchers’ constructions of autistic people as sub-human, or as objects, may facilitate this harm.

Previously, McGuire (2016) highlighted that the abstraction of autism from individual and into an amorphic form might facilitate the acceptance and normativity of violence again autistic people. This has also been discussed by autistic scholars (Farahar, 2022) who highlight that the abstraction distracts from the fact that in talking about “autism,” we are inherently talking about autistic people. This abstraction has been highlighted by autistic academics when discussing why person-first language might be problematic (Botha et al., 2021). In the qualitative accounts provided, our participants relied on a semantic separation of autism, potentially because preventing “autism” sounds less problematic than preventing autistic people. This fits with the notion raised by McGuire (2016) that by using abstraction, society can “combat,” “defeat,” “eradicate,” without sounding like material harm is coming to any one person.

Importantly, language cannot be relied upon by researchers who fail to see the fundamental humanness of autistic people. While person-first language (“person with autism”) is touted as an answer to remembering the fact that autistic people are human too, when coupled with sentiments which deny the personhood, autonomy, or humanness of autistic people, it is still dehumanization, objectification, or stigmatization, but with extra semantic steps. Conversely, coupling identity-first language and “neurodiversity-lite” (den Houting, 2019; Chapman, 2021) or basing someone’s value on normativity, a bell-curve of intelligence, and degrees of independence still constitutes dehumanization, objectification, and eugenics. The adoption of identity-first language means very little if next you are denying autistic people complex emotions, identity, community, culture, and objectifying and othering them.

### Limitations

This study has several limitations and strengths. This study used a novel technique for identifying ableism and narratives in researcher’s accounts of autistic people. If we were to use traditional and well-known measures of stigma (such as social distance scales, e.g., Gillespie-Lynch et al., 2015), researchers may be more likely to display demand characteristics in line with social desirability. By using open-ended questions, we elicited spontaneously driven narratives, which may more closely reflect participants actual beliefs. However, a limitation is that this process has a higher degree of subjectivity around what constitutes medicalized, or social narratives, as well as ableism, and participants likely still shared somewhat socially desirable answers. A further limitation is the low response rate to our targeted emails (7%)—and many of our respondents were from Psychology and researching topics that may align more with the priorities of autistic people, compared to what traditionally gets funded (e.g., Pellicano et al., 2014). Therefore, the views expressed are unlikely to be representative of the entire industry of autism research. Nonetheless, it is valuable to have obtained and analyzed the perspectives of almost 200 autism researchers, and to contribute to a discussion on the future of autism research.

### Reflection

Subjectivity is inherently a part of all autism research, as researchers have an undeniable power in terms of framing, study design, and representation of data, regardless of whether this goes acknowledged by researchers (Botha, 2021). Acknowledgment of positions, and disruptive reflexivity can be key to challenging the *status quo* (Pillow, 2003). Further, Pillow (2003), p. 193) pushes for messy reflexivity which “leave us in the uncomfortable realities of doing engaged qualitative research.”

Those who subscribe to positivistic paradigms may come to this section looking for comfort or reassurance about the validity and generalizability of this study, and what was done to factor in our own biases. While we took steps to make this a rigorous study—including basing the content analysis coding framework on extant definitions of phenomena, working reflexively and collaboratively to make decisions, actively acknowledging our values and discussing disagreements—the process of research *is* complex and messy. Our research will be “contaminated” by personal experience in ways which will make people uncomfortable, regardless of what we say to inspire trustworthiness or rigor. In terms of representation, it is a piece of research conducted collaboratively by both an autistic and non-autistic researcher, on autism researchers (some of whom were autistic, but the majority were non-autistic), on their perception of autistic people and autism research.

As lead author (MB)—I am autistic, I have been constructed by autistic researchers my whole life—I was diagnosed in their legacy, and the legacy of their research has become a way of constituting me, including my dehumanization. There is a process of reversal here, where I am granted some power to do the same in return—I am writing them, as they are writing me. Even when I create work which is less “messy,” I am relegated in knowledge creation about autism by virtue of my autisticness (Botha, 2021). But here, I am tasked with representing the people who have grown accustomed to both representing me and denying me my own ability to represent myself. Many may be unhappy with the representations we have made, regardless of how close we have positioned this work to their words or actions.

What then, becomes of the non-autistic autism researchers? As one participant expressed, they felt that they were being “pushed” out of the field. Much of what we have written in this paper will cause discomfort to non-autistic autism researchers. As a non-autistic autism researcher (EC) I have grown increasingly concerned about the state of autism research and my own position within it. Even in what is a small sample of self-selecting people, there are red flags everywhere. It can feel like those taking an anti-ableist stance are in a minority. And even then, is the stand being taken enough? Perhaps discomfort must be felt—as the first step to sitting up and realizing that change is needed. The last thing needed is more division—rather than feeling pushed out, *feel pulled in*. The emotional burden should not be placed all on the autistic autism researchers—non-autistic autism researchers should actively work toward carrying the load too. The discomfort you feel is probably only a tiny fraction of the pain that has been felt by autistic people reading your papers.

### Conclusion

In this mixed-method study we aimed to understand the narratives researchers had of autistic people and autism research, and whether involving autistic people in autism science might relate to a lower odds of cues of ableism in researchers’ narratives. There was a relationship between involving autistic people in autism research and lower odds of ableist cues in researchers’ narratives about autistic people, though we cannot posit a direction to this relationship (it may well be reciprocal). While some of the results could be considered bleak (including the ways in which autistic people are dehumanized), there is still hope for the future. The disruption of ableism in autism research is key to the development of rigorous research, and findings demonstrate that meaningful inclusion of autistic people matters. Further, there were examples of researchers actively working to resist, reframe, and challenge ableism in research. These findings point to a hopeful future. Future research should aim to identify good practice for involving autistic people, the conditions needed to produce equitable involvement of autistic people in autism science, and the direction of such relationships.

## Data availability statement

The raw data supporting the conclusions of this article will be made available by the authors, without undue reservation.

## Ethics statement

The studies involving human participants were reviewed and approved by University of Stirling General University Ethics Panel. The participants provided their written informed consent to participate in this study.

## Author contributions

This research was a part of MBs fellowship research which was designed in collaboration with EC. Both authors contributed to study design, data collection, data analysis, and writing up.
